# Effects of Predation by Protists on Prokaryotic Community Function, Structure, and Diversity in Anaerobic Granular Sludge

**DOI:** 10.1264/jsme2.ME16067

**Published:** 2016-07-12

**Authors:** Yuga Hirakata, Mamoru Oshiki, Kyohei Kuroda, Masashi Hatamoto, Kengo Kubota, Takashi Yamaguchi, Hideki Harada, Nobuo Araki

**Affiliations:** 1 Department of Civil Engineering, National Institute of Technology, Nagaoka CollegeNagaoka, NiigataJapan; 2 Department of Science of Technology Innovation, Nagaoka University of TechnologyNagaoka, NiigataJapan; 3 Department of Environmental Systems Engineering, Nagaoka University of TechnologyNagaoka, NiigataJapan; 4 Department of Civil and Environmental Engineering, Tohoku UniversitySendai, MiyagiJapan; 5 New Industry Creation Hatchery Center (NICHe), Tohoku UniversitySendai, MiyagiJapan

**Keywords:** anaerobic protozoa, up-flow anaerobic sludge blanket (UASB) reactor, anaerobic granular sludge

## Abstract

Predation by protists is top-down pressure that regulates prokaryotic abundance, community function, structure, and diversity in natural and artificial ecosystems. Although the effects of predation by protists have been studied in aerobic ecosystems, they are poorly understood in anoxic environments. We herein studied the influence of predation by *Metopus* and *Caenomorpha* ciliates—ciliates frequently found in anoxic ecosystems—on prokaryotic community function, structure, and diversity. *Metopus* and *Caenomorpha* ciliates were cocultivated with prokaryotic assemblages (*i.e.*, anaerobic granular sludge) in an up-flow anaerobic sludge blanket (UASB) reactor for 171 d. Predation by these ciliates increased the methanogenic activities of granular sludge, which constituted 155% of those found in a UASB reactor without the ciliates (*i.e.*, control reactor). Sequencing of 16S rRNA gene amplicons using Illumina MiSeq revealed that the prokaryotic community in the UASB reactor with the ciliates was more diverse than that in the control reactor; 2,885–3,190 and 2,387–2,426 operational taxonomic units (>97% sequence similarities), respectively. The effects of predation by protists in anaerobic engineered systems have mostly been overlooked, and our results show that the influence of predation by protists needs to be examined and considered in the future for a better understanding of prokaryotic community structure and function.

Predation by protists is a major cause of prokaryotic attrition in natural and artificial ecosystems (53) and modulates prokaryotic abundance and community function, structure, and diversity. The impact of protist bacterivory has been well documented in aerobic ecosystems (57), in which protists consume up to 100% of the prokaryotic standing stock each d (2, 3, 5). Protists show species-specific prey preferences (26, 41, 66), which result in the disappearance of specific prokaryotes in ecosystems (49, 60, 68) as well as an increase or decrease in community diversity (7, 56, 67). Furthermore, predation by protists increases microbial activities including bacterial growth (9, 67). In contrast, limited information is available on the influence of protist predation on anoxic ecosystems (44, 52, 64), except for the rumen. In the rumen, predation by protists increases or decreases the richness of archaeal species (51); one study showed that predation influences prokaryotic abundance and methanogenic activities, but not community diversity (46). The protists *Metopus* and *Caenomorpha* are free-living ciliates frequently found in anoxic freshwater (11, 44), marine sediments (18, 78), landfill sites (21, 24), rice field soil (64), and wastewater treatment processes (31). However, the impact of predation by these genera on prokaryotic community structure and diversity in anoxic ecosystems, particularly engineered ecosystems, has been poorly characterized.

The long-term cocultivation of ciliates and their prey (*i.e.* prokaryotic assemblages) is essential for investigating the influence of protist predation on anoxic ecosystems because the rates of consumption of prokaryotic standing stocks are generally low in anoxic ecosystems (less than 0.1–6%) (23, 44, 61). The batch cultivation method has been traditionally used in the anoxic cultivation of ciliates (50, 80); however, this method has an intrinsic limitation: the predator-to-prey ratio changes transiently during the cultivation. Additionally, most studies on batch cultivation have involved artificial substrates other than prokaryotic cells (*e.g.*, wheat powder) to maintain the growth of ciliates (50); therefore, it is not possible to assess the influence of predation by protists. In order to overcome these issues, a few research groups have attempted to cultivate protists using the continuous cultivation method (9, 56, 67); however, the successful cultivation of *Metopus* or *Caenomorpha* ciliates for >100 d in a continuous cultivation system has not yet been achieved.

Although the impact of predation by protists on prokaryotic community structure and diversity has been studied by DNA fingerprinting including denaturing gradient gel electrophoresis and terminal restriction fragment length polymorphism (7, 46, 49, 51,) in addition to fluorescence *in situ* hybridization (25, 34, 60), the resolution of these methods only allows researchers to analyze differences in major prokaryotic populations. Thus, alterations in the structure and diversity of a prokaryotic community induced by protist predation have not yet been assessed in detail. The sequencing of 16S rRNA gene amplicons using Illumina MiSeq or HiSeq recently became the standard method for an in-depth analysis of the microbial-community structure because this analysis yields >10,000 sequence reads for each sample and allows researchers to examine community structures in detail (12). These methods have also recently been applied to follow changes in prey communities upon predation (6).

Consequently, our objectives were 1) to cocultivate *Metopus* and *Caenomorpha* ciliates and prokaryotic assemblages in a continuous cultivation system and 2) to study the influence of predation by these protists on prokaryotic community function, structure, and diversity in anaerobic engineered ecosystems, in particular, in an up-flow anaerobic sludge blanket (UASB) reactor. A UASB reactor that was packed with granular sludge (*i.e.*, prokaryotic assemblages) was operated for 171 d on domestic sewage. The proliferation of *Metopus* and *Caenomorpha* ciliates was verified microscopically and by sequencing the 18S rRNA gene amplified by single-cell PCR. In order to assess the effects of predation by the *Metopus* or *Caenomorpha* ciliates, another UASB reactor served as a control reactor, in which their proliferation was inhibited by cycloheximide, a specific growth inhibitor for eukaryotes. After 171 d of operation, the sequencing of 16S rRNA gene amplicons using Illumina MiSeq was performed, and prokaryotic community structures and diversities were compared between the two UASB reactors.

## Materials and Methods

### Inoculums

Granular sludge was collected from a UASB reactor (1148 L; 4 m in height, 0.56 m in diameter) at a domestic wastewater treatment plant (Nagaoka, Japan). The reactor had been continuously and stably operated for more than five years on domestic sewage; the operational conditions and chemical oxygen demand (COD) removal efficiency were described previously (71). Ciliates were detected in inoculums using optical microscopy, and their abundance was less than 10 cells mL^−1^.

### Operation of laboratory scale UASB reactors

**Fig. 1:**
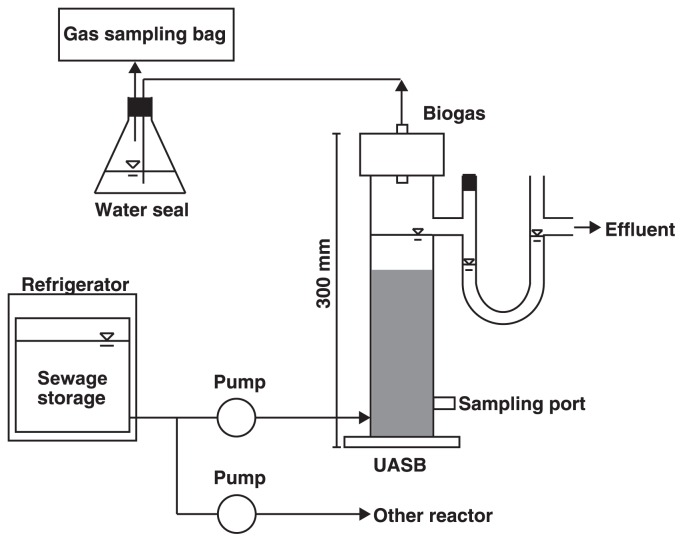
A schematic diagram of an up-flow anaerobic sludge blanket (UASB) reactor. Sewage was supplied from a single storage bottle to 2 UASB reactors (*i.e.*, the coculture and control reactors) by peristaltic pumps. The UASB reactor configuration was the same between the 2 reactors.

Two UASB reactors (500 mL; 6 cm in diameter and 30 cm in height; Fig. 1) were operated for 171 d after an inoculation with 400 mL of granular sludge. The UASB reactors were operated with the continuous feeding of domestic sewage collected from a domestic wastewater treatment plant (Nagaoka, Japan). The typical composition of sewage is shown in Table S1. Prior to use, sewage was filtered through a polyethylene sieve (mesh size of 350 μm) in order to remove large solid particles. The approximate loading rates of total COD were increased in a stepwise manner by shortening the hydraulic retention time (HRT) as follows: 0.5 (g COD) L^−1^ d^−1^ with 10 h of HRT for 0–83 d (Run 1), 1.0 (g COD) L^−1^ d^−1^ with 5 h of HRT for 83–138 d (Run 2), 2.5 (g COD) L^−1^ d^−1^ with 2 h of HRT for 138–160 d (Run 3), and 4.0 (g COD) L^−1^ d^−1^ with 1.25 h of HRT for 160–171 d (Run 4). The reactors were operated in an isothermal room at 20±2°C. The control of pH was not used during the operation, and the pHs of the influent and effluent were 7.1±0.2 (average ± standard deviation) and 6.7±0.2, respectively. The biogas generated in the UASB reactors was collected in a gas-sampling aluminum bag (Techno Quartz, Tokyo, Japan) installed on top of the reactors. Granular sludge was collected from a sampling port located 3 cm above the bottom of the reactors.

In one UASB reactor, the proliferation of protists was inhibited by the addition of cycloheximide (1 g L^−1^) at the start of operation (day 0), as described previously (32, 35). Cycloheximide is an antibiotic that is specific for eukaryotes and binds to the initiation factor of the eukaryotic 60S ribosomal subunit, thereby inhibiting protein synthesis in eukaryotic cells (63). Cycloheximide has traditionally been employed to prepare prokaryotic cultures without eukaryotes (32, 33, 35, 58). Although the growth inhibition of anaerobic bacteria by cycloheximide was previously reported (30, 75), the addition of cycloheximide did not influence bacterial or methanogenic activities in an anaerobic reactor (58). Supplementation with cycloheximide was repeated from day 40 to day 70 of the operation every ten day (Run 1′) because the protists were detectable in the control reactor on day 40 of the operation. The UASB reactors with and without the cycloheximide treatment were hereafter called the control and coculture reactors, respectively.

### Chemical analysis

pH levels were measured using the pH meter D-51 (Horiba, Kyoto, Japan). Total and soluble COD concentrations were determined using HACH option 435 on a DR-2000 spectrophotometer (Hach, Tokyo, Japan) according to the manufacturer’s instructions. In order to quantify the soluble COD fraction, liquid samples were filtered through GB-140 glass filter paper (pore size of 0.45 μm; Advantec, Tokyo, Japan), and the filtrates were subjected to COD measurements.

Suspended solid (SS) and retained sludge (mixed liquor suspended solids: MLSS) concentrations were measured in accordance with the standard method (4). The sludge volume in the UASB reactors was calculated from the retained sludge concentrations and sludge bed volume.

Gas composition was measured by injecting 1 mL of a gas sample into a GC-2014 gas chromatograph (Shimadzu, Kyoto, Japan) equipped with a thermal conductivity detector and molecular sieve-5A column (Shimadzu). The retention times of methane, hydrogen, and carbon dioxide gases were measured by analyzing standard gases purchased from GL Science (Tokyo, Japan). The volume of the gas that was collected in a gas sampling bag was assessed by the liquid displacement method using 0.5N NaOH. Methanogenic activities were calculated from the volumes of daily methane gas production and amounts of anaerobic granular sludge retained in UASB reactors.

### Analysis of protozoan community structure

The abundance of ciliates was determined manually by counting the numbers of ciliate cells in a Neubauer chamber (ERMA, Tokyo, Japan) under an IX71 light microscope (Olympus, Tokyo, Japan). At least five randomly selected visual fields were used for counting. *Metopus* and *Caenomorpha* ciliates were identified according to their morphological features as reported previously by Esteban *et al.* (18) and Martin-Gonzalez *et al.* (40), respectively. The phylogenetic affiliation of the ciliates was further ascertained by sequencing the 18S rRNA gene. After 171 d of operation, stand-alone cells of the *Metopus* and *Caenomorpha* ciliates were physically isolated using MM-89 and IM-9B micromanipulators (Narishige, Tokyo, Japan). After several washes, each cell was transferred into a sterile PCR tube containing 3 μL of sterile distilled water. The cell was disrupted by three freeze-thaw cycles (*i.e.*, freezing at −80°C and thawing at 60°C) and was then directly subjected to PCR. The oligonucleotide primers Euk-82F (5′-AAACTGCGAATGGCTC-3′) and MedlinB (5′-TGATCCTTCTGCAGGTTCACCTAC-3′) (39, 45) were used to amplify a region of the eukaryotic 18S rRNA gene. The PCR reaction mixture had a volume of 10 μL and contained the oligonucleotide primers (0.5 μM each), deoxynucleoside triphosphates (dNTPs; 200 μM each), 1× PCR buffer, and AmpliTaq Gold (0.025 U μL^−1^; ThermoFisher Scientific, Yokohama, Japan). PCR was performed using a C1000 thermal cycler (Bio-Rad Laboratories, Benicia, CA, USA) under the following cycling conditions: at 95°C for 10 min; 50 cycles at 95°C for 45 s, at 56°C for 45 s, and at 72°C for 2 min; with a the final extension step at 72°C for 10 min. The amplification of the 18S rRNA gene region was ascertained by agarose gel electrophoresis using DNA Size Marker 4 (Nippongene, Tokyo, Japan). The amplicon was purified using a Gene Clean Turbo Kit (Qiagen, Hilden, Germany), and DNA was sequenced by the Sanger method using a 3730xl DNA Analyzer (Thermo Fisher Scientific, Yokohama, Japan). The nucleic acid sequences obtained were aligned in Clustal W software and a phylogenetic tree was constructed in MEGA 6.06 software (72) by means of the maximum likelihood (ML; Jones-Taylor-Thornton model), neighbor joining (NJ; Poisson model), maximum parsimony (MP; close neighbor interchange in the random-tree search algorithm), and unweighted pair group methods with an arithmetic mean (UPGMA; a maximal composite likelihood model) using the 18S rRNA gene of *Discophrya collini* (GenBank accession number L26446) as an outgroup.

### Analysis of bacterial and archaeal community structures

Genomic DNAs were extracted from granular sludge using an ISOIL Beading Kit for Beads (Nippongene). Genomic DNA extraction was replicated from granular sludge in both reactors. The concentrations of the extracted DNAs were measured by means of the Picogreen dsDNA Quantification Kit (Thermo Fisher Scientific) and a Versafluor fluorometer (Bio-Rad Laboratories, Benicia, CA, USA). Amplification of the 16S rRNA gene region was performed using the oligonucleotide primers 515F (5′-GTGCCAGCMGCCG CGGTAA-3′) and 806R (5′-GGACTACHVGGGTWTCTAAT-3′) corresponding to the V4 region of the 16S rRNA gene (13). The PCR mixture had a volume of 50 μL and contained 50 ng of the extracted DNAs, the oligonucleotide primers (0.5 μM each), dNTPs (200 μM), 1×PCR buffer, and AmpliTaq Gold (0.025 U μL^−1^). The cycling conditions were as follows: at 94°C for 3 min; 30 cycles at 94°C for 45 s, followed 50°C for 1 min, then 72°C for 1 min 30 s; and finally 72°C for 5 min. The amplicon was purified and used in the preparation of a library by means of MiSeq Reagent Kit v2 nano (Illumina, San Diego, CA, USA) for sequencing on Illumina MiSeq. Amplicon library concentrations were measured using BioAnalyzer DNA 1000 (Agilent Technologies, Santa Clara, CA, USA). The quality of the sequencing analysis was verified by examining a PhiX library prepared from a PhiX spike-in control (Illumina). Sequence reads with a low quality score (Phred quality score ≤30) were eliminated using the fastx_trimmer tool, and paired-end sequence reads were then assembled in the paired-end assembler for the Illumina sequence software package (PANDAseq) (0). Nucleic acid sequences with ≥97% similarity were grouped into an operational taxonomic unit (OTU) by the UCLUST algorithm (17).

The phylogenetic affiliations of the OTUs were identified using a blastn search against reference sequences in the Greengenes database, version 13_5 (16) and the nr database (National Center for Biotechnology Information). A phylogenic tree was constructed as described above, and the 16S rRNA gene of *Methanopyrus kandleri* (GenBank accession number AB301476) was used as an outgroup. Species richness estimates Chao1 and phylogenetic diversity (PD) were calculated using Quantitative Insight into Microbial Ecology (QIIME) software, version 1.7.0. (12). The Simpson index—meaning species evenness—was also calculated in QIIME. A principle component analysis (PCA) and Welch’s *t*-test were performed using STAMP software (54).

### Accession numbers

The partial 18S rRNA gene sequences of *Metopus* sp. and *Caenomorpha* sp. were deposited in the GenBank/EMBL/DDBJ databases under the accession numbers LC027270 (1,190 bp) and LC027271 (1,195 bp). The bacterial and archeal 16S rRNA gene sequences are available under accession numbers AB938329 to AB948126 and LC152435 to LC152737.

## Results

### Reactor performance

The two UASB reactors were operated in parallel with the continuous feeding of domestic sewage for 171 d. The coculture and control reactors showed similar COD and SS removal efficiencies (Table 1); *i.e.*, the average total COD and SS removal efficiencies were >57% and >85%, respectively. The methanogenic activities of the coculture reactor from Run 2 to Run 4 were significantly higher (*P*<0.05, Student’s *t*-test) than those of the control reactor. Activities increased at higher COD loading rates and reached 7.3±0.7 and 4.7±1.5 mL (g sludge)^−1^ d^−1^ (average ± standard deviation) in the coculture and control reactors, respectively, during Run 4.

### Population dynamics of *Metopus* and Caenomorpha ciliates

**Fig. 2:**
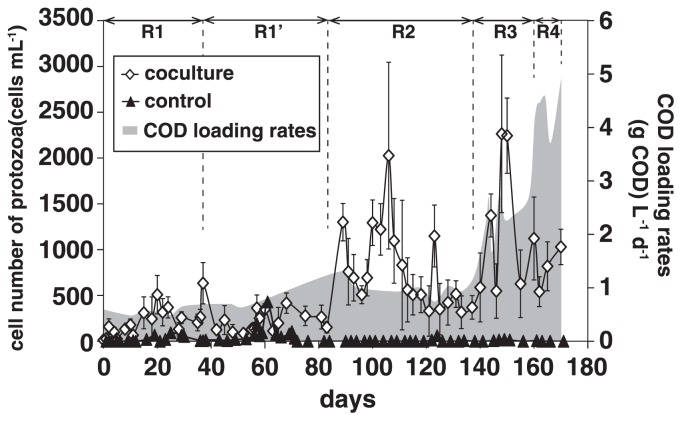
Abundance of *Metopus* and *Caenomorpha* ciliates and total chemical oxygen demand (COD) loading rates in up-flow anaerobic sludge blanket (UASB) reactors: R1, Run 1; R1′, Run 1′; R2, Run 2; R3, Run3; and R4, Run4. Error bars represent the standard deviation.

**Fig. 3:**
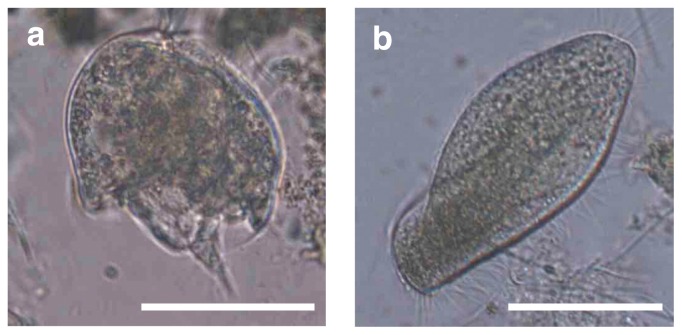
Microscopy of *Caenomorpha* (panel a) and *Metopus* (panel b) ciliates found in the UASB reactor. The scale bar is 50 μm.

**Fig. 4:**
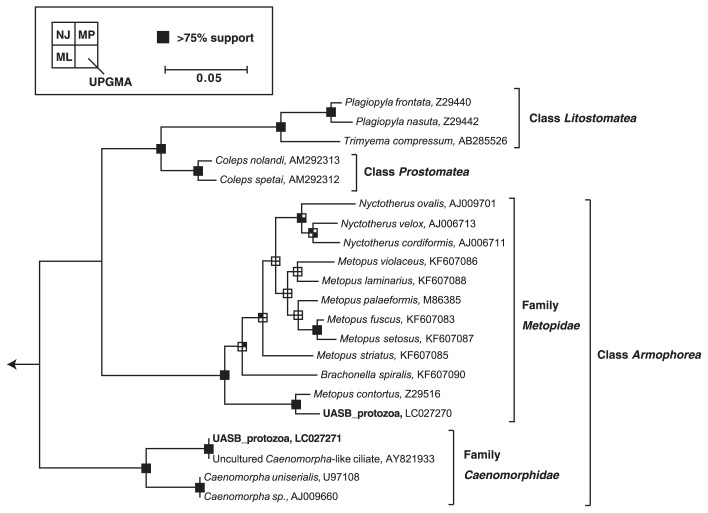
An 18S-rRNA based neighbor joining (NJ) tree showing the phylogenetic affiliation of *Metopus* and *Caenomorpha* ciliates found in an up-flow anaerobic sludge blanket (UASB) reactor. Branching points that support a probability >75% in the bootstrap analyses (based on 1,000 replicates, estimated using the NJ method, maximum likelihood method [ML], maximum parsimony method [MP], and unweighted pair group method with an arithmetic mean [UPGMA]) are shown as black squares. The scale bar represents 5% sequence divergence.

The abundance of ciliates in the coculture reactor increased at higher COD loading rates (Fig. 2). In the coculture reactor, two types of morphologically different ciliates were found under an optical microscope (Fig. 3a and b). They were swimming around granular sludge and predating microbial cells (Movie. S1). Based on their morphological features, those protists were identified as *Metopus* sp. and *Caenomorpha* sp.; *Caenomorpha* ciliates accounted for 80% of the total population of ciliates. The proliferation of protists other than *Metopus* sp. and *Caenomorpha* sp. was not found in the coculture reactor. Phylogenetic affiliations were further analyzed by examining 18S rRNA gene sequences amplified from physically isolated *Metopus* and *Caenomorpha* ciliate cells. The 18S rRNA gene sequences that were obtained from *Metopus* and *Caenomorpha* ciliate cells were affiliated with the family *Metopidae* and *Caenomorphidae*, respectively (Fig. 4); the sequence similarity of the 18S rRNA gene to *Metopus contortus* was 95% (accession number Z29516) and to an uncultured *Caenomorpha*-like ciliate was 97% (AY821933). The *Caenomorpha* ciliates contained endosymbiotic methanogens (*i.e.*, *Methanobacterium* sp.) according to F_420_-fluorescence (Fig. S1) and 16S rRNA gene amplicon sequencing data (Table S2) (77); this result is in agreement with previous findings, which also showed the presence of endosymbiotic methanogens in *Caenomorpha* ciliates (23).

### Structure of bacterial and archaeal communities

These structures were examined in seeding anaerobic granular sludge (0 d) and sludge collected from the coculture and control reactors after 171 d of reactor operation by sequencing PCR-amplified 16S rRNA gene regions using Illumina MiSeq. Two replicate libraries were prepared from each sample, and a total of 14,367 to 32,809 valid bacterial and archaeal sequences were recovered (Table 2). The sequences were clustered into OTUs (≥97% sequence similarity), and community structures in each sludge sample were compared by PCA (Fig. S2). The PCA analysis indicated that shifts in community structure occurred during the 171 d of reactor operation. The number of OTUs increased from 0 d to 171 d of reactor operation, and a larger number of OTUs were found in the coculture reactor than in the control reactor: 2,885–3,190 and 2,387–2,426 OTUs, respectively (Table 2). The values of the species richness estimates Chao1 and PD were also greater in the coculture reactor, indicating that the bacterial and archaeal community structures in the coculture reactor were more diverse than those in the control reactor.

**Fig. 5:**
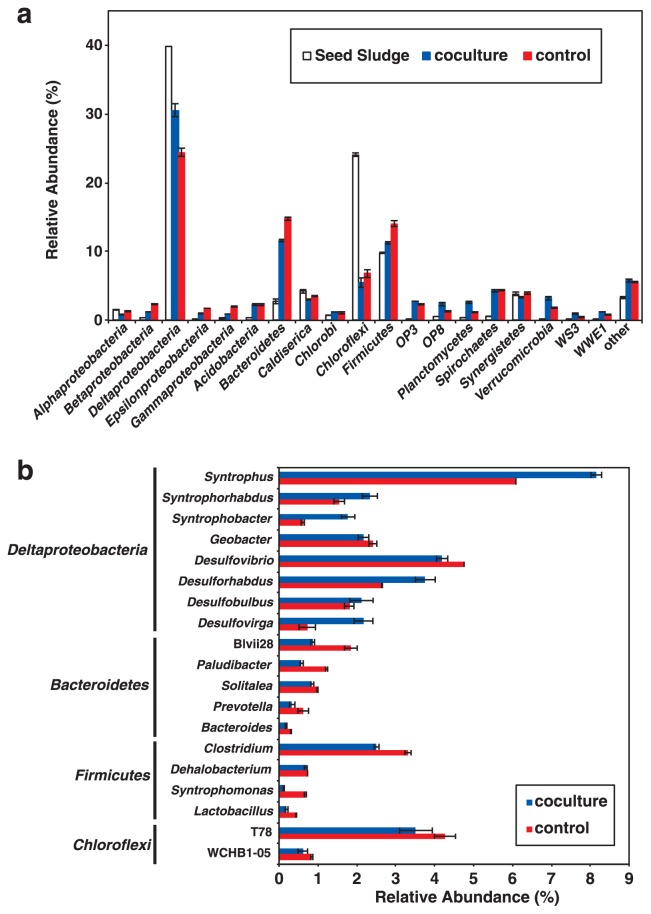
Taxonomic classification of bacterial communities in anaerobic granular sludge collected after 171 d of operation of an up-flow anaerobic sludge blanket (UASB) reactor. (a) Abundance of 16S rRNA gene sequences from each bacterial phylum and class. Sequence reads that are not classified into any known bacterial group were labeled as “other”. Approximately 4% of all reads were archeal 16S rRNA gene sequences, which are not shown in this figure (but are shown in Fig. 6a). (b) Abundance of 16S rRNA gene sequences from each bacterial genus in major bacterial groups (the class *Deltaproteobacteria* and phyla *Bacteroidetes*, *Firmicutes*, and *Chloroflexi*). Coculture and control: UASB reactors with and without *Metopus* and *Caenomorpha* ciliates, respectively.

Bacteria accounted for 96% of the total number of sequences, and the taxonomic classification of the bacterial communities was shown in Fig. 5a. The class *Deltaproteobacteria* and phyla *Bacteroidetes* and *Firmicutes* dominated both systems, while relative abundance was different between the samples; *i.e.*, *Deltaproteobacteria* were more abundant, while *Bacteroidetes* and *Firmicutes* were less abundant in the coculture reactor. The relative abundance of the following bacterial genera differed between the control and coculture reactors: *Syntrophus*, *Syntrophorhabdus*, *Syntrophobacter*, *Desulforhabdus*, *Desulfovirga* (class *Deltaproteobacteria*), *Paludibacter*, OTU-Blvii28 (phylum *Bacteroidetes*), and *Clostridium* (phylum *Firmicutes*) (Fig. 5b).

**Fig. 6:**
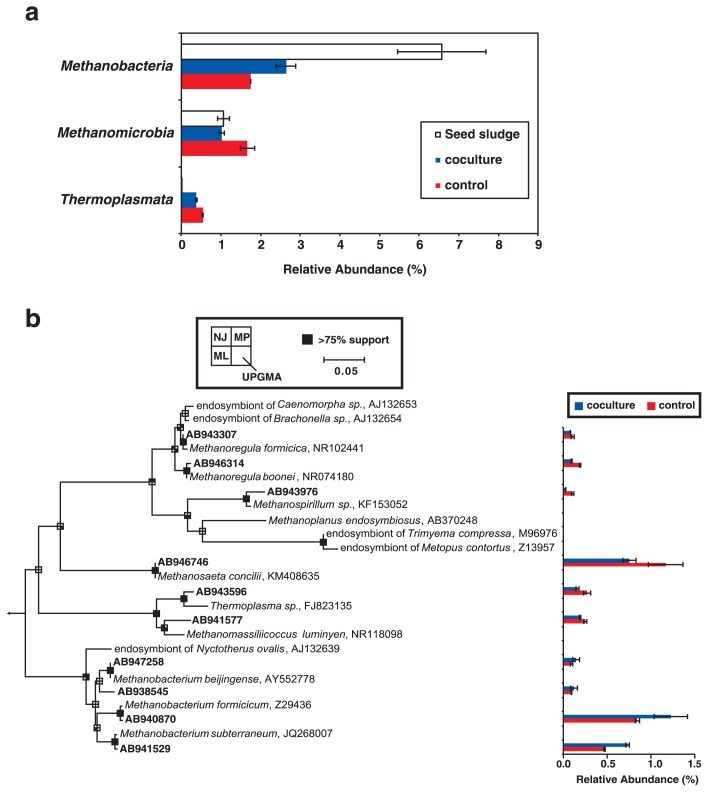
Taxonomic classification of archaeal communities in anaerobic granular sludge collected after 171 d of operation of an up-flow anaerobic sludge blanket (UASB) reactor. (a) Abundance of 16S rRNA gene sequences from each archaeal class. Coculture and control: UASB reactors with and without *Metopus* and *Caenomorpha* ciliates, respectively. (b) A 16S rRNA-based neighbor-joining (NJ) tree showing the phylogenetic affiliations of archaeal operational taxonomic units (OTUs) in the UASB reactors. Boldface indicates OTUs found in the present study. The scale bar represents the substitution of 5% of bases. Branching points that support probability >75% in the bootstrap analyses (based on 1,000 replicates, estimated using the NJ method, maximum likelihood method [ML], maximum parsimony method [MP], and unweighted pair group method with an arithmetic mean [UPGMA]) are shown as black squares. The abundance of sequence reads that are affiliated with each OTU is shown in the graph on the right.

The taxonomic classification of the archaeal community structures was shown in Fig. 6a. All sequence reads were affiliated with the archaeal classes *Methanobacteria*, *Methanomicrobia*, or *Thermoplasmata* in the phylum *Euryarchaeota*. The classes *Methanobacteria* and *Methanomicrobia* were abundant in both systems, while the class *Methanobacteria* was more abundant in the coculture reactor than in the control reactor. A phylogenetic analysis of archaeal 16S rRNA gene sequences indicated that the genus *Methanobacterium* were more abundant, while *Methanosaeta* were less abundant in the coculture reactor (Fig. 6b).

## Discussion

The cocultivation of ciliates and prokaryotic assemblages is the first step during an analysis of the impact of predation by protists, and long-term cocultivation is necessary for assessing this impact because the rates of consumption of prokaryotic standing stocks by ciliates are generally low in anoxic ecosystems (23, 44, 61). *Metopus* ciliates were previously detected in a UASB reactor processing diluted waste-water (1); this finding is suggestive of the suitability of a UASB reactor as a cultivation tool for these ciliates. In the present study, it was demonstrated that a UASB reactor fed sewage allows for the cocultivation of *Metopus* and *Caenomorpha* ciliates and prokaryotic assemblages for 171 d. Notably, *Caenomorpha* ciliates have been detected in a wide range of natural freshwater systems (23, 28, 44), whereas the cultivation of *Caenomorpha* ciliates has rarely been described. UASB reactors have an excellent capacity for biomass retention (36); this characteristic may allow for the proliferation of slow-growing *Metopus* and *Caenomorpha* ciliates; the doubling time of these ciliates in the coculture reactor were roughly estimated to be 2.5–5 d, judging by the increase in their cell numbers after 96–106 d and 137–148 d (Fig. 2). In addition, sulfide-rich sewage (4.3 mg-S L^−1^) (*i.e.*, 0.13 mM); Table S1) was fed into the UASB reactors; this may have resulted in the selective growth of *Metopus* and *Caenomorpha* ciliates. These ciliates prefer sulfide-rich (>1 mM) ecosystems (23, 43, 78), whereas other anaerobic ciliates are sensitive to sulfide (44); *e.g.*, the minimum inhibitory concentration of sulfides for *Coleps* ciliates is 0.01 mM (55). In the present study, *Metopus* and *Caenomorpha* ciliates were cultivated in the range of 10^2^ to 10^3^ cells mL^−1^, which is higher than that in a freshwater lake (<10^2^ cells mL^−1^), but smaller than that in the rumen (10^5^ cells mL^−1^) (15, 29, 44).

Methane gas production was more prominent in the coculture reactor; this result is consistent with previous findings (8, 32). In these studies, an increase in the specific microbial activities of methanogenesis and sulfate reduction was found in anoxic ecosystems with protists. Although *Caenomorpha* ciliates contain endosymbiotic methanogens (Fig. S1), the contribution of symbionts to the increase in methanogenic activities is expected to be minor. If the abundance of the ciliates and endosymbiotic methanogens and specific methanogenic activities by the symbionts are assumed to be 2,500 ciliates mL^−1^ (Fig. 2), 4,500 cells ciliates^−1^ (64, 77), and 0.97 (fmol methane) endosymbiont^−1^ h^−1^ (23), respectively, the contribution to methane production during Run 3 is estimated to be less than 0.1%. Instead of the symbiotic contribution, the *Metopus* and *Caenomorpha* ciliates may have stimulated microbial activities in our experiments through the decomposition of organic material and processing of minerals, as demonstrated previously for aerobic protists (9, 20). It is important to note that total COD and SS removal efficiencies were similar between the coculture and control reactors, while methane gas production was greater in the coculture reactor (Table 1). The greater production of methane gas in the coculture reactor may have resulted from the degradation of particular COD fractions to soluble organic matter by the ciliates (50). Additionally, hydrogenotrophic methanogens (*i.e.*, *Methanobacterium*) were more abundant, while acetotrophic methanogen (*i.e.*, *Methanosaeta*) were less abundant in the coculture reactor (Fig. 6b). The greater abundance of hydrogenotrophic methanogens in the coculture reactor may have contributed to an increase in methanogenic activities in the coculture reactor because hydrogenotrophic methanogens showed higher methanogenic activities than those of acetotrophic methanogens (70). Methane gas production and its recovery in UASB reactors have been implemented for the use of methane gas as an alternative energy source to fossil fuels. An increase in methanogenic activities is desirable for this purpose, and the mechanisms involved need to be examined in more detail in future studies

The sequencing of 16S rRNA gene amplicons using Illumina MiSeq was performed in order to investigate the influence of predation by *Metopus* and *Caenomorpha* ciliates on prokaryotic community structure and diversity. Community structure and diversity were investigated in seeding anaerobic granular sludge and sludge collected from the coculture and control reactors after 171 d of reactor operation. Our outcomes showed that predation by *Metopus* and *Caenomorpha* ciliates resulted in alterations in the microbial community structure and diversity. This result was consistent with previous findings showing that predation by aerobic ciliates enhanced community evenness and diversity (7, 62). Although grazing by the *Metopus* and *Caenomorpha* ciliates on prokaryotic assemblages was not measured directly in this study, all of the ciliates contained food vacuoles packed with prokaryotic cells, as assessed by optical microscopy, strongly suggesting that the ciliates were consuming prokaryotic cells. The amount of the microbial biomass retained in a UASB reactor was smaller in the coculture reactor than in the control reactor (Table 1); this phenomenon may have resulted from predation by the protists.

The abundance of the 16S rRNA gene sequences of *Paludibacter*, OTU-Blvii28, and *Clostridium* was lower in the coculture reactor; this effect may have resulted from selective grazing by the *Metopus* and *Caenomorpha* ciliates. These ciliates were swimming around prokaryotic assemblages (*i.e.*, granular sludge; S1 Movie) and consuming microbial cells; therefore, the microbial cells located in the outer layer of granular sludge were preferentially eaten by the ciliates. The outer layer of granular sludge in the UASB reactor was dominated by bacterial cells and performed the function of the initial anaerobic degradation of complex organic compounds to simpler ones (19, 65). Bacteria affiliated with the genera *Paludibacter*, OTU-Blvii28 (*Bacteroidetes*) (69, 76), and *Clostridium* (*Firmicutes*) (14, 38) were rod- or coccus-shaped bacteria producing extracellular hydrolase and contributed to anaerobic degradation. These bacteria were previously detected in the outer layer of granular sludge in a UASB reactor, as identified using a fluorescence *in situ* hybridization analysis (19, 37, 74). On the other hand, filamentous bacteria affiliated with the phylum *Chloroflexi* were also previously found in the outer layer of granular sludge in a UASB reactor. In the present study, the 16S rRNA gene sequences of OTU-T78 and OTU-WCHB1-05 showed 91% and 92% sequence similarities to the 16S rRNA gene sequence of *Leptolinea tardivitalis* (accession number; NR_040971), a known filamentous bacterium affiliated with the phylum *Chloroflexi* (81–83). As shown in Fig. 5b, the abundance of 16S rRNA sequences affiliated with OTU-T78 and OTU-WCHB1-05 was not significantly different between the coculture and control reactors. This result suggests that *Metopus* and *Caenomorpha* ciliates selectively grazed rod or coccus-shaped bacterial cells located in the outer layer.

The 16S rRNA gene sequences of *Syntrophus*, *Desulfovirga*, *Syntrophobacter*, and *Syntrophorhabdus* were more abundant in the coculture reactor (Fig. 5b). These bacteria prefer volatile fatty acids as a carbon source (10, 47, 59, 73), whereas the concentrations of these fatty acids were always below the detection limit (<0.1 mM) in both UASB reactors in our study. *Metopus* (18, 78) and *Caenomorpha* (22) ciliates have a unique organelle, the hydrogenosome, instead of mitochondria, in which organic matter is oxidized to volatile fatty acids (*i.e.*, acetate, valeric acid, and lactic acid) and hydrogen for ATP synthesis (27, 48, 84). The *Metopus* and *Caenomorpha* ciliates may have produced volatile fatty acids in the hydrogenosome, thereby ensuring the greater proliferation of *Syntrophus*, *Desulfovirga*, *Syntrophobacter*, and *Syntrophorhabdus* in the coculture reactor. Additionally, the hydrogenosome of *Metopus* and *Caenomorpha* ciliates produces hydrogen, which enabled the greater proliferation of hydrogenotrophic methanogens (*i.e.*, *Methanobacterium*) in the coculture reactor (Fig. 6b), as demonstrated previously (31, 48, 79). Very limited information is available on the underlying symbiotic associations between ciliates and prokaryotic assemblages in UASB reactors, and specific interactions between the ciliates and *Syntrophus*, *Desulfovirga*, *Syntrophobacter*, *Syntrophorhabdus*, and *Methanobacterium* need to be examined in future studies.

Predation by *Metopus* and *Caenomorpha* ciliates altered the prokaryotic community structure, diversity, and function (*i.e.* methanogenesis) in our UASB reactors. *Metopus* and *Caenomorpha* ciliates have been found in a wide range of anoxic ecosystems including anoxic freshwater (11, 44), marine sediments (18, 78), landfill sites (21, 24), and rice field soil (64). Predation by protists needs to be taken into consideration in the future in order to obtain a better understanding of prokaryotic ecology in these anoxic ecosystems.

## Supplementary Information



## Figures and Tables

**Table 1:** Performance of the coculture reactor and control reactor. Two up-flow anaerobic sludge blanket (UASB) reactors—with and without ciliates (*i.e.*, coculture and control reactors, respectively)—were operated for 171 d.

period (d)	reactor	sample (*n*)	COD removal		SS removal (%)	methane production mL (g sludge)^−1^ d^−1^	retained sludge (g L^−1^)

			total (%)	soluble (%)			
Run 1 0–37	coculture control	*n*=15	75 ± 7	52 ± 9	97 ± 2	n.d.	38.5
			73 ± 7	51 ± 8	97 ± 1	n.d.	

Run 2 83–138	coculture control	*n*=19	73 ± 13	51 ± 17	97 ± 2	1.4 ± 1.1	31.5
			67 ± 13	50 ± 16	95 ± 3	0.6 ± 0.5	35.2

Run 3 139–159	coculture control	*n*=16	64 ± 16	48 ± 11	95 ± 4	3.5 ± 1.2	31.1
			61 ± 21	46 ± 12	93 ± 5	2.3 ± 0.9	33

Run 4 160–171	coculture control	*n*=12	67 ± 14	50 ± 6	92 ± 7	7.3 ± 0.7	27.6
			57 ± 19	47 ± 10	85 ± 16	4.7 ± 1.5	31.2

COD: chemical oxygen demand, SS: suspended solids, n.d.: not determined. Data are presented as the mean ± standard deviation.

**Table 2:** Community richness, diversity, and evenness indices of anaerobic granular sludge collected from the coculture reactor and control reactor. Anaerobic granular sludge was collected after 171 d of operation, and two replicate libraries were prepared for each sludge sample.

	Number of sequence reads	OTUs	coverage	Chao1	PD	Simpson
seed sludge (*n*=2)	14,367	1,600	0.92	8,914	115	0.91
	14,422	1,436	0.93	7,526	107	0.91

coculture (*n*=2)	32,809	3,134	0.95	9,498	204	0.99
	27,870	2,839	0.94	8,127	191	0.99

control (*n*=2)	28,277	2,372	0.96	5,052	162	0.99
	30,378	2,321	0.96	4,717	158	0.99

OTU: operational taxonomic unit (≥97% sequence similarity), PD: phylogenetic diversity.
