# Supplementary material for: Effects of Predation by Protists on Prokaryotic Community Function, Structure, and Diversity in Anaerobic Granular Sludge
Source: Microbes Environ. 2016 Jul 12;31(3):279–87. doi: 10.1264/jsme2.ME16067 (PMC5017804; doi:10.1264/jsme2.ME16067)
Supplement: Supplementary file 1 [file 31_279_s2.pdf]

Supplemental information

This section is composed of two table, two figures, and one movie file.

Supporting Table

Table S1: Composition of the sewage that was fed into up-flow anaerobic sludge blanket (UASB) reactors in the present study. COD_{Cr}: chemical oxygen demand determined using a dichromate, BOD_5: biological oxygen demand for 5 d, SS: suspended solids, VSS: volatile suspended solids, TKN: total Kjeldahl nitrogen, T-N: total nitrogen, and T-P: total phosphorus. The soluble fraction was prepared by filtration through the glass fiber membrane GB-140. The data are presented as mean ± standard deviation.

COD_{Cr} (mg L^{-1})		BOD_5 (mg L^{-1})		SS	VSS	Sulfide
Total	Soluble	Total	Soluble	(mg L^{-1})	(mg L^{-1})	(mg S) L^{-1}
269 ± 73	122 ± 34	182 ± 92	87 ± 37	80 ± 37	71 ± 27	4.3 ± 0.7

SO_4^{2-} (mg S) L^{-1}	NH_4^+ (mg N) L^{-1}	NO_3^- (mg N) L^{-1}	NO_2^- (mg N) L^{-1}	TKN (mg N) L^{-1}	T-N (mg N) L^{-1}	T-P (mg P) L^{-1}
14 ± 17	21 ± 6	0.03 ± 0.05	0.18 ± 0.17	29 ± 5	29 ± 5	3.5 ± 0.7

Table S2: Taxonomic classification of the prokaryotic communities in *Caenomorpha* ciliate cell. A single *Caenomorpha* ciliate cell was separated after 171 d of operation using a micromanipulator, and the prokaryotic 16S rRNA gene sequence was amplified by PCR using oligonucleotide primers 515F and 806R as previously described (Hirakata et al., 2015). The amplified 16S rRNA gene sequence was determined using the MiSeq sequencer (Illumina, San Diego, CA, USA), and the sequence data were analyzed using

OTU	Accession number	Relative abundance	Closely related species (accession number)	Domain	Sequence similarity (%)
denovo1	LC152435	89.7%	*Methanobacterium* sp. (KJ432636.1)	Archaea	100%
denovo2	LC152436	3.6%	*Lactobacillus sakei* (KT968365.1)	Bacteria	100%

the QIIME software (version 1.8.0). OTU: operational taxonomic unit (≥97% sequence similarity). OTUs that abundance was less than 1% were grouped and labeled as “other”. *n.a*: not applicable.

denovo3	LC152437	1.7%	*Escherichia coli* (KU161315.1)	Bacteria	100%
other	*n.a*	5.0%	*n.a*	*n.a*	*n.a*

Reference

Hirakata, Y., M. Oshiki, K. Kuroda, M. Hatamoto, K. Kubota, T. Yamaguchi, H. Harada and N. Araki. 2015. Identification and detection of prokaryotic symbionts in the ciliate *Metopus* from anaerobic granular sludge. Microbes Environ. 30: 335-338.

Supporting Figure Legends

Figure S1: Endosymbiotic methanogens in *Caenomorpha* ciliates. Left: F_{420} fluorescence, right: a phase contrast image. The scale bar is 100 µm.

Figure S2: Comparison of the prokaryotic community structures found in seeding anaerobic granular sludge (square) and the sludge collected from coculture and control reactors after 171 d of operation (triangle and circle, respectively). Principle component analysis (PCA) of community structures at operational taxonomic units (OTU) (≥97% sequence similarity) level.

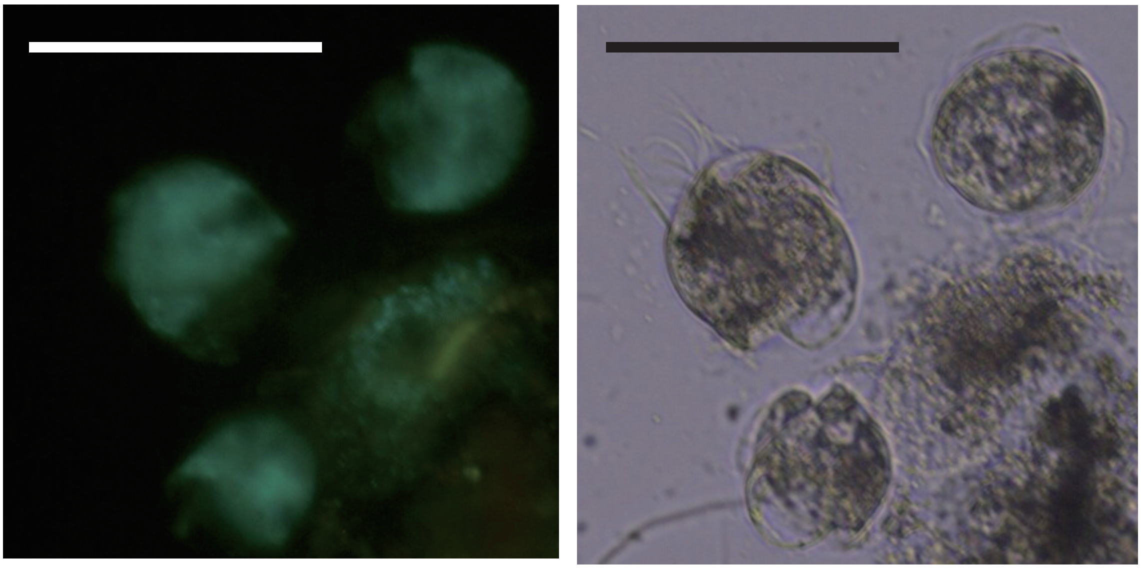

Fig. S1 (Hirakata et al., 2016)

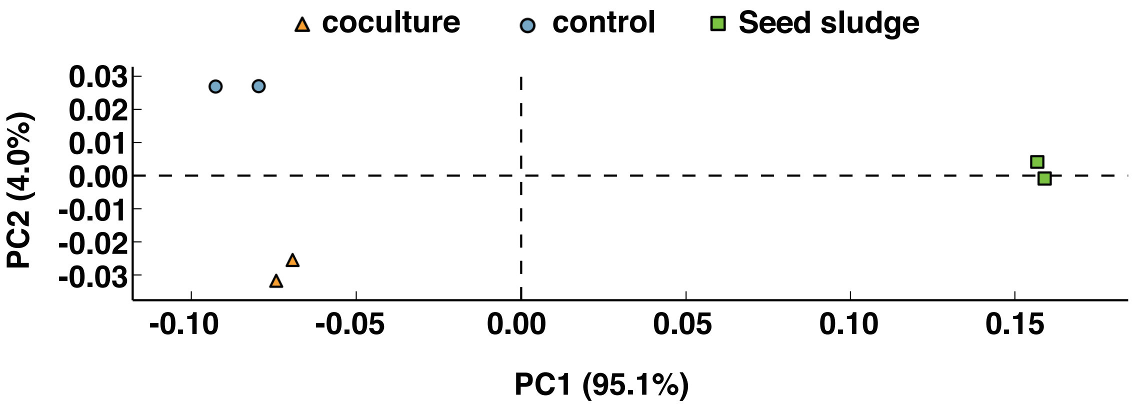

Supplementary Fig.S2 (Hirakata et al., 2016)
